# Work Ability Index of Shift Working Hospital Nurses in Jordan

**DOI:** 10.2174/1874434601812010116

**Published:** 2018-06-29

**Authors:** Dalky F. Heyam, Gharaibeh Besher, Al-Khateeb Nesreen

**Affiliations:** Faculty of Nursing, Jordan University of Science & Technology, P.O Box 3030, Irbid 22110, Jordan

**Keywords:** Work ability, Job satisfaction, Shift work, Nurses, Jordan, Work Ability Index, Mueller/McCloskey Satisfaction Scale

## Abstract

**Background:**

Despite the extensive literature on work ability, few studies have looked at variables associated with work ability of nurses working fixed versus rotating shifts.

**Objective:**

The study aims were to explore variables contributing to work ability and to examine the association of demographic, job satisfaction, and work shift to work ability.

**Method:**

A cross-sectional design was utilized to assess work ability level and job satisfaction among nurses working 8 or 12 hour rotating or fixed shifts in Jordanian hospitals. The data collection tools were the Work Ability Index and the Mueller/McCloskey Satisfaction Scale. Data were analyzed to determine the extent to which job satisfaction, shift work, and demographic variables were associated with work ability.

**Results:**

Work ability level was “moderate”, while job satisfaction level was “moderately dissatisfied”. A positive significant relationship was found between work ability and job satisfaction (r = 0.347, n = 349, p < 0.000). This relationship was higher for fixed-shift workers (r =.507) compared to rotating-shift workers (r = .299). Standard linear multiple regression analysis indicated that job satisfaction level predicted work ability level (β =.347, p = .000).

**Conclusion:**

The study confirmed that promoting job satisfaction leads to higher work ability, and thus, enhances the quality of care provided. The finding that job satisfaction is predictive of work ability has implications for training intervention.

## 1 INTRODUCTION

Worldwide, nurses are the largest workforce in the health care system and are in need of good physical and mental health abilities to perform their job effectively [1, 2]. These physical and mental abilities are the main components of the concept of work ability [3]. Work ability affects a person’s ability to deal with working life, work demands, and job performance. Work ability has been reported in literature for various occupations [2], however; the concept has been little explored in relation to nurses working in fixed and rotational shifts.

The concept of work ability was developed in 1980 by the Finnish Institution of Occupation Health (FIOH). Work ability refers to how well the workers are able to perform their work [4]. Furthermore, the FIOH developed the Work Ability Index **(**WAI) to measure perceptions of workers regarding their physical, mental, and social health and their ability to cope with job demands [4]. Since then, the WAI has been translated into 21 languages and used in studies concerning work ability levels among workers of various occupational backgrounds [1].

Work ability is an essential concept to assess and promote among nurses working in a hospital environment. Promoting work ability is likely to improve nurses’ work effectiveness and enhance job satisfaction, which, in turn, improves overall health care quality [5]. Conversely, low work ability leads to negative impact on work effectiveness and job satisfaction. The results of a study of 4306 nurses in 10 public and private hospitals in Europe were that low WAI scores were associated with work dissatisfaction, work conditions, lack of time for sports or leisure time, and low social support from colleagues [6]. Further, the results of a systematic review noted that factors associated with low WAI scores included lack of relaxation time, poor musculoskeletal ability, older age, obesity, lack of autonomy, elevated mental work demands, poor work environment, and elevated physical effort load [3]. Although the review reported results for healthcare workers, factors such as job satisfaction and shift work demands were not studied.

Nurses are among the many healthcare professionals affected by the requirement to work outside of normal daytime hours, as in working alternate shifts [7]. This may lead to long working hours (12 vs. 8 hours) and non-ergonomic planning of work schedules (*e.g*. lack of rest time between shifts and working consecutive night shifts or weekends.). Hence, difficulties in shift-work-related problems, both for the nurses and for those supervising them, may increase the current global nursing shortage [8].

Shift work is a known cause of disturbances in the health and well-being of nurses [9]. However, nursing services must be available on a 24-hour basis, making shift work a necessity. The effect of shift work on nurses’ lifestyle, their occupational health issues, and the demands of staffing are documented [8]. Also, adverse effects have been noted on workers’ physiological, psychological, and health related problems due to an impairment of biorhythms, although adverse effects vary according to type of shift worked, as in rotational versus fixed shifts [10].

It was reported that staff working rotational shifts experienced poor work ability, which caused high distress among working staff [11, 12]. In another study, nurses who worked longer shifts (as in 12 hour vs. 8 hour shifts) were susceptible to burnout and made frequent errors at work [11]. Also, the authors reported that nurses with low WAI scores reported lower job satisfaction [11]. Hence, identifying factors that could influence work ability, such as job satisfaction and type of shift worked, is crucial to maintain nursing efficiency and to promote and achieve health care goals.

In Jordan, there is little literature concerning the effect of job satisfaction of nurses working standard shifts (fixed shift) or non-shifts (rotating shift) on work ability. Although studies conducted in Jordan have investigated job satisfaction [13, 14], no study was found that specifically addressed work ability and its association with job satisfaction among nurses working fixed or rotating shifts. The current study was designed to fill this gap and provide baseline information regarding work ability among nurses working varying shifts in Jordanian teaching hospitals. For purposes of this study, shift work refers to either rotating or fixed shift work. In the hospitals from which the sample was drawn, nurses worked either 8 or 12 hour rotating day or night shifts, or they worked 8 or 12 hour morning, evening, or night fixed shifts.

### 1.1 Aims of the Study

The primary aim of this study was to examine level of work ability among hospital nurses in Jordan. The secondary study aims were as follows:

1. Determine level of work ability and job satisfaction between nurses working rotating or fixed shifts.
2. Identify associations between work ability and job satisfaction for nurses working four types of shifts (8 hour rotating, 12 hour rotating, 8 hour fixed, 12 hour fixed).
3. Determine whether demographic factors are associated with or predict work ability or job satisfaction.

## 2 MATERIALS AND METHODS

### 2.1 Research Design and Sample

A cross-sectional design was used to assess levels of work ability and job satisfaction for nurses working in two teaching hospitals in Jordan. The target population consisted of nurses working all shifts across all departments of the teaching hospitals who were available at the time of data collection. The inclusion criteria were registered nurses who worked rotational or fixed shifts with at least one year of work experience. Nurse Managers or supervisors were not included due to differing work hours and roles. To gain an estimate for sample size, G* power [15] for a medium effect size at an 0.05 alpha level at 80% power resulted in 350, to which 50 was added to account for attrition, bringing the total estimated account to 400. However, 390 data collection sets were returned and due to incomplete data, 41 were rejected. This resulted in a final sample size of 349, and time restrictions did not allow for followup of the 41 respondents to increase the sample size. The response rates from the two hospitals were as follows: 40% (n = 160) from one hospital and 47.9% (n = 190, although 1was withdraw) from the other.

### 2.2 Measures

Respondents completed a data sheet for identifying personal demographics and type of shift worked. Respondents were asked to state their age, gender, marital status, educational level, years of work experience, family status, type of the work setting (intensive care, medical-surgical, obstetric, pediatric, or emergency) and type of shift work. In this study, the shifts worked were fixed 8 hour shifts (morning, evening, or night), 12 hour shifts (day or night), rotating 8 hour shifts (morning, evening or night) or rotating 12 hour shifts (day or night).

The data collection instruments used in this study were Arabic versions of the Work Ability Index (WAI) and the Mueller/McCloskey Satisfaction Scale (MMSS). Agreements were obtained to translate the instruments into Arabic. Arabic versions of both instruments were developed using a translation and back translation technique. Performed at a Certified Language Center, the English versions were translated into Arabic and then back translated into English by a different language expert. A panel of professional bilingual and bicultural nursing professionals assessed the Arabic versions for content validity. A pilot test of the Arabic versions (n=40) resulted in Cronbach’s alpha coefficients of 0.85 for the WAI and 0.95 for the MMSS.

The WAI, a widely used index of work ability, measures respondents’ perceptions regarding their physical, mental, and social health and their ability to cope with job demands [4]. The questions cover current work ability, ability to work within job demands, the number of illnesses/limitations, impairments affecting work ability, the amount of sick leave taken within the last year, and an estimate of work ability within 2 years’ time. As each answer has a different score, the total work ability score is calculated by adding scores across the dimensions. The resulting score range is 7 (low work ability) to 49 (high). The authors reported a Cronbach’s alpha coefficient of 0.83 and a content validity index of 0.79 [4].

The MMSS, originally developed for hospital nurses, measures job satisfaction. The scale contains 31 items, and each item is scored on a 5-point Likert scale, where, 1 corresponds to very dissatisfied, 2 to moderately dissatisfied, 3 to neither satisfied nor dissatisfied, 4 to moderately satisfied, and 5 to very satisfied. The MMSS evaluates job satisfaction in relation to satisfaction with extrinsic rewards, scheduling, family/work balance, co-workers, interaction, professional opportunities, praise/recognition, and control/responsibility. The alpha coefficient for the scale is 0.89 and the content validity index is 0.83 [16].

### 2.3 Data Collection Procedure

Approval to conduct the study was obtained from the Institution Review Board (IRB) of the principle investigator’s academic institution and administrators of the target hospitals. Times of data collection covered all shift options to ensure nurses had an equal chance to participate in the study. To recruit nurse respondents, all nurses available at time of data collection were given a cover letter that explained the purpose of study and ensured them that participation or non-participation would not be reported to their supervisors. The nurses were informed that participation was voluntary, and respondents’ anonymity and information confidentiality were ensured. Nurses interested in participating signed a consent form. Nurses were given the study questionnaires (demographic form, WAI, and MMSS) in an enclosed envelope. They completed the instruments on site and handed them back to the researchers.

### 2.4 Data Analysis

The data were analyzed using the Statistical Package for Social Sciences (SPSS; IBM Statistics for Macintosh, Version 22.0). Descriptive statistics permitted describing sample characteristics. Pearson correlation coefficient tests were used to identify the relationship between work ability and job satisfaction. Independent sample t-tests were used to compare mean differences in work ability and job satisfaction for rotational versus fixed shift workers. Multiple regression tests were used to identify possible predictors of work ability. Although the sample size of 349 was short of the 400 (350 + 50 for attrition) estimated to achieve power, results can be considered indicative, yet must be treated with caution.

## 3 RESULTS

### 3.1 Sample Characteristics

The mean age of participants was 29 years, with an average of 6 years of work experience, which was deemed adequate to provide work experiences for assessing work ability and job satisfaction. Although female nurses outnumbered male nurses (206 to 143), the difference was 63 less males than females. All but two nurses (diploma) had baccalaureate or higher degrees (n=347, 99.4%). More than half of nurses (n=221, 63.4%) worked in 8 hour rotating shifts (morning, evening or night), with the smallest frequency of the sample working 12 hour fixed shifts (n = 28, 8.0%). As nurses working in all shifts within the hospital at the time of data collection were included in the sample, their work settings were distributed across hospital departments. All but two of the nurses worked as staff nurses. More than half of the participants (n= 204, 58.3%) lived in a city other than that of the hospital’s location. Most of participants reported that transportation was not provided by their hospitals (n=263, 75.1%). Table **1** shows sample characteristics according to demographic variables.

### 3.2 Work Ability and Job Satisfaction

The total mean WAI score among hospital nurses was moderate (µ = 29.3, SD = 6.8). For total WAI scores, the classification by range of scores is as follows: Low, 7-27; Moderate, 28-33; Good, 34-43; and Very good, 44-49. The highest scoring dimension subscale was “estimate of work ability as compared to job demand” (µ = 5.84, SD =1.89), where the score range was 2-10. The lowest scoring dimension subscale was number of current diseases/limiting conditions (µ = 2.61, SD = 2.25), where the score range was 1-7.

The total mean score for MMSS was low (µ = 34.6, SD = 8.50), equivalent to moderately dissatisfied. For total MMSS scores, the range is 31 (very dissatisfied) to 155 (very satisfied). The dimension subscale with the highest mean score was “satisfaction with external rewards” (µ = 1.8, SD = .82), where the range was 1-5. The subscale dimension with the lowest mean score was “satisfaction with work/family balance” (µ = .74, SD = .88), where the range was 0-4.

### 3.3 Work Ability among Shift Workers and Non-Shift Workers

For purpose of this study, shift working was defined as the type of shift the nurse worked, which for participating nurses was fixed shifts (8 hours or 12 hours) corresponding to “shift worker” designation, or was rotating (8 hours or 12 hours), corresponding to “non-shift worker” designation. An independent sample t-test showed no significant difference (t = -1.37, p > 0.05) between mean WAI scores for shift workers (M = 29.5, SD = 6.5) and non-shift workers (M = 28.3, SD = 8). However, a one way ANOVA showed a significant difference in the levels of work ability between the 4 types of fixed and rotating shifts (F = 11.17, p < .01). A *post hoc* Scheffe test was used to determine the difference between these shifts. The test showed a mean difference in work ability between fixed 8 hour shifts and 12 hour rotating shifts, but not a mean difference between fixed 12 hour shifts and rotating 12 hour shifts, as shown in Table **2**.

### 3.4 Relationships among Study Variables

A correlational matrix was constructed using Pearson’s product- moment correlation to assess to what extent demographic variables and work shift correlated with work ability (WAI scores) and job satisfaction (MMSS scores). The matrix used Pearson correlation for continuous variables and point biserial correlation for dichotomous variables. The continuous independent variable that significantly correlated to work ability was “work experience in years” (r =^-^.311, p <.001). Categorical variables that significantly correlated to work ability were “being married” (r =^-^ .254, p < .001) and “transportation unavailable “(r= .212, p <.001). The correlational matrix is shown in Table **3**.

To identify the relationship between work ability and job satisfaction; the results revealed a positive, significant relationship between WAI and MMSS mean scores (r =.347, n = 349, p < 0.000). The higher level of work ability correlated with a higher level of job satisfaction. Moreover, the results revealed a positive, significant relationship for rotating shift workers (r = .299, n=282, p < .000), albeit the correlation between rotating shifts and WAI scores was not strong. A stronger relationship was noted for fixed-shift workers (r =.507, n=67, p < .000).

### 3.5 Work Ability Predictors

Standard linear multiple regression analysis was used to identify significant predictors of nurses’ work ability. The data were assessed for outliers, multicollinearity, normality, linearity, and independence of residuals. In relation to WAI scores, where the variables showing significance (demographic, work shift and job satisfaction) were used in the regression analysis, results indicated that 44.4% of work ability variance was explained by the independent variables (R^2^ =.444, adjusted R^2^=.395), and the model was significant (F = 9.08, p < .000). Of the demographic variables that were significantly correlated with work ability, only “years of work experience” and “marital status” did not significantly predict work ability (t= 0.3 and t=.09 respectively). In addition, a significant relationship was found between job satisfaction and work ability when accounting for demographic variables (B = .1, t = 5.2, p < .01). The linear multiple regression analysis results in relation to WAI scores are shown in Table **4**.

In relation to MMSS scores, or job satisfaction, the regression analysis showed that work ability significantly influenced job satisfaction (B = .7, t = 5.2, p < .000). The regression model showed that the factors significantly predicted job satisfaction (F = 12.2, p < .01) and accounted for 20% of the variability of the job satisfaction scores (R^2^ = 0.201). When nurses reported high job satisfaction level, they experienced high work ability level. The linear multiple regression analysis results in relation to MMSS scores are shown in Table **5**.

## 4 DISCUSSION

The study was designed to add knowledge of factors contributing to optimizing productivity in the context of work ability of rotating and fixed shift nurses in Jordanian public hospitals and the relationship of job satisfaction to work ability. Results of the current study indicated that nurse respondents self-reported a moderate work ability level (WAI total score mean of 29.3). This result was lower than found in a study of Taiwanese nurses, where the reported a WAI mean score was 36.4 (in the Good range), which the authors considered to be an “average” work level [17]. However, authors at an academic medical center in the Netherlands [18] reported a considerably lower mean of 8 (in the Low range). Given this diversity, additional studies are needed to investigate which factors contribute most and least to nurses’ work ability and establish how scores relate to low, moderate, or high levels of work ability.

Job satisfaction is one aspect associated with work ability and needs to be considered as a contributing factor. In the current study, nurses with higher job satisfaction scores (MMSS highest score highest was 93 of 155) also had higher work ability scores (the highest WAI was 46 of 49). Similar results were found in a study of nurses in an emergency unit; that is, when job satisfaction increased, work ability increased [19]. In the current study, results indicated that Jordanian nurses in teaching hospitals were moderately dissatisfied with their job. In other studies of job satisfaction [20, 21], Jordanian hospital nurses rated their job satisfaction as neutral (“neither satisfied or dissatisfied”). However, one study found that Jordanian mental health workers rated their job satisfaction as moderate [13].

Possible causes of less than high job satisfaction may be due strict organizational policies and lack of effective communication between medical doctors and nursing staff, especially in teaching hospitals. In addition, nurses in teaching hospitals face job stressors, such as increased workloads, and having to perform multiple non-nursing roles, such as clerk, educator, family mediator, or student preceptor. In addition, the Syrian crisis is likely influencing job satisfaction because of the increased pressure on hospitals resources to care for refugees.

### 4.1 Work Ability and Shift Work

In terms of the relationship between shift work and work ability, few studies have examined how one influenced the other. One study reported that nurses who worked fixed night shift had WAI scores that were significantly lower than nurses who worked rotating day or night shifts [22]. Supporting this, another study reported that night shift work was associated with low work ability scores [23]. Further, a third study reported that there was negative relationship between shift work and work ability for nursing personal [24]. However, in contrast, a fourth study reported no association between shifts’ worked and work ability [25].

Possible contributions to inconsistencies in research findings may be because participants differed in the type of nursing work performed. More so than time or type of shift, differing work settings have differing protocols, work procedures, and socio-cultural expectations.

The current study is the first to investigate work ability level among shift work nurses in Jordan. Additional studies are needed to determine other factors that contribute to and affect work ability among nurses. Focus should be on differences between work ability level for shift workers and non-shift workers, factors that predict work ability level, and the association of job satisfaction for shift workers with work ability. In addition, longitudinal studies are needed to determine how favorable work conditions help to maintain and improve work ability.

## CONCLUSION

This study is an early effort to assess work ability level and factors contributing to it among nurses in Jordan. A main finding is that job satisfaction is predictive of work ability. Additionally, the type of work shift, albeit a possible contributor needs definitive study. Although it is beyond the purpose of this study, an implication is that low work ability could have negative outcomes for nurses working in a hospital environment. The potential for negative outcomes deriving from low work ability should be taken into account when planning program interventions to improve nursing efficiency. Therefore, the current study has implications for practice, policymaking, and research. Nurse administrators in cooperation with Nursing Councils should aid in developing workplace policies to enhance work ability and, therefore, job satisfaction, especially for nurses working 12-hour rotating shifts.

### STUDY LIMITATIONS

As an initial investigation of associations of work ability for shift working nurses in Jordan, the study served as a one-time study of nurses’ self-reported perceptions. A longitudinal study would produce a more comprehensive overview of existing relationships, especially as self-reporting is itself subjective, and could help in establishing causality. The sample respondents were recruited from teaching hospitals, which may have stricter policies and procedures than other general acute care hospitals. In addition, the sample size, although near to, did not reach the estimated sample size to achieve power.

## Figures and Tables

**Table 1:** Descriptive characteristics of nurse participants (n = 349).

Participants' Characteristics*	*F*requency (%)	*M* (SD)	Range
Age		29.2(5.2)	22-46
Work Experience in years		6.01(4.3)	1-26
Gender - Male - Female	143 (41.0%) 206 (59.0%)	–	–
Marital Status - Single - Married/Other	160 (45.8%) 189 (54.2%)	–	–
Educational Level - Diploma - Baccalaureate - Master/Doctoral degree	2 (.6%) 312 (89.4%) 35 (10%)	–	–
Presence of children in home - Yes - No	140(40.1%) 209 (59.9%)	–	–
Working Shift - 8 hour rotating shift - 12 hour rotating shift - 8 hour fixed shift - 12 hour fixed shift	221 (63.3%) 61 (7.4%) 39 (11.2%) 28 (8%)	–	–
Work Setting - Emergency - Pediatrics - Maternity and delivery - Medical/surgical ward - Operation/recovery - Intensive care units - Out-patient clinics/Other	27 (7.7%) 69 (19.8%) 52 (14.9%) 73 (20.9%) 35 (10%) 82 (23.5%) 11 (3.1%)	–	–
Living Place* - Same city as workplace - Other city than workplace	144 (41.1%) 203 (58.9%)	–	–
Transportation - Provided by hospital - Not Provided by hospital	87 (24.9%) 262 (75.1%)	–	–

*Note: Data reflects participants with valid data on the variable.

**Table 2:** Scheffe test assessment of differences in work ability between types of shifts worked.

Dependent Variable (I) work shift	(J) work shift	Mean Difference (I-J)	Std. Error	Significance
Fixed 8 hour	rotating 8 hour fixed 12 hours rotating 12 hour	-.99321 3.26099 3.98172*	1.14044 1.62645 1.34622	.859 .261 .034
Rotating 8 hour	fixed 12 hours rotation 12 hours	4.25420* 4.97493*	1.31716 .94968	.016 .000
fixed 12 hour	rotating 12 hour	.72073	1.49887	.972

* Significant at the 0.05 level.

**Table 3:** Pearson product-moment correlation coefficients of demographic variables with WAI total score, MMSS total score, and work shift.

Variable	WAI Total Score	MMJS Total Score	Work Shift
Age	-.370**	-.307**	-.309**
Work experience (yrs.)	-.313**	-.190**	-.315**
Gender	.043	-.060	-.082
Marital status	-.141**	-.061	-.166**
Degree level	.028	.023	-.020
Work shift	-.255**	-.025	.264**
Work setting	.070	.104	-.009
Transportation	-.211**	-.161**	-.029
Living Place	.054	-.087	.121*
Work ability	1	.347**	.074
Job satisfaction	.347**	1	-.002

** Correlation is significant at the 0.01 level (2-tailed).

* Correlation is significant at the 0.05 level (2-tailed).

**Table 4:** Standard linear multiple regression analysis of demographic, work shift, and WAI variables.

		Coefficients^a^				
Model		Unstandardized Coefficients		Standardized Coefficients	t	Sig.
		B	Std. Error	Beta		
1	(Constant)	39.067	3.255		12.002	.000
	Age	- .304	.125	-.234	- 2.436	.015*
	Work experience (yrs.)	- .045	.146	-.029	- .309	.757
	Marital status	- .065	.703	-.005	-.092	.926
	Work setting	- 1.722	.739	-.125	-2.330	.020*
	Work shift	- 1.223	.409	-.159	-2.991	.003*
	Transportation	- 2.004	.769	-.127	-2.605	.010*
	Total MMJS score	.101	.019	.260	5.213	.000*

^a^Dependent variable: Total WAI score

* Significant at the 0.05 level (2-tailed).

**Table 5:** Standard linear multiple regression analysis of demographic. work shift, and MMSS variables.

		Coefficients^a^					
Model			Unstandardized Coefficients		Standardized Coefficients	t	Sig.
			B	Std. Error	Beta		
1	(Constant)		46.630	10.114		4.610	.000
	Age		-1.436	.329	-.431	-4.369	.000**
	work experience in years		1.046	.388	.260	2.696	.007*
	Marital status		1.865	1.885	.054	.990	.323
	place of work		4.620	1.985	.131	2.328	.021*
	working shift		.057	1.112	.003	.052	.959
	transportation		-1.857	2.084	-.046	-.891	.374
	WAI total score		.730	.140	.284	5.213	.000**

Dependent Variable: total MMSS score

** Significant at the 0.01 level (2-tailed).

* Significant at the 0.05 level (2-tailed).
